# Explaining variance in perceived research misbehavior: results from a survey among academic researchers in Amsterdam

**DOI:** 10.1186/s41073-021-00110-w

**Published:** 2021-05-03

**Authors:** Tamarinde Haven, Joeri Tijdink, Brian Martinson, Lex Bouter, Frans Oort

**Affiliations:** 1 Department of Philosophy, Vrije Universiteit, Amsterdam, De Boelelaan 1105, 1081 HV Amsterdam, The Netherlands; 2 Department of Ethics, Law and Humanities, Amsterdam UMC, Vrije Universiteit Amsterdam, De Boelelaan 1117, Amsterdam, Netherlands; 3 Department of Research, HealthPartners Institute, 8170 33rd Ave. S., Bloomington, MN 55425 USA; 4 Center for Care Delivery and Outcomes Research, Minneapolis Veterans Affairs Health Care System, One Veterans Drive, Minneapolis, MN 55417 USA; 5 Department of Medicine, University of Minnesota, 420 Delaware St SE, Minneapolis, MN 55455 USA; 6 Department of Epidemiology and Data Science, Amsterdam UMC, Vrije Universiteit Amsterdam, De Boelelaan 1117, Amsterdam, The Netherlands; 7 Research Institute of Child Development and Education, University of Amsterdam, Nieuwe Achtergracht 127, 1018 WS Amsterdam, The Netherlands

**Keywords:** Research climate, Research misbehavior, Publication pressure, Research integrity

## Abstract

**Background:**

Concerns about research misbehavior in academic science have sparked interest in the factors that may explain research misbehavior. Often three clusters of factors are distinguished: individual factors, climate factors and publication factors. Our research question was: to what extent can individual, climate and publication factors explain the variance in frequently perceived research misbehaviors?

**Methods:**

From May 2017 until July 2017, we conducted a survey study among academic researchers in Amsterdam. The survey included three measurement instruments that we previously reported individual results of and here we integrate these findings.

**Results:**

One thousand two hundred ninety-eight researchers completed the survey (response rate: 17%). Results showed that individual, climate and publication factors combined explained 34% of variance in perceived frequency of research misbehavior. Individual factors explained 7%, climate factors explained 22% and publication factors 16%.

**Conclusions:**

Our results suggest that the perceptions of the research climate play a substantial role in explaining variance in research misbehavior. This suggests that efforts to improve departmental norms might have a salutary effect on behavior.

**Supplementary Information:**

The online version contains supplementary material available at 10.1186/s41073-021-00110-w.

## Background

There has long been concern about research misbehavior in academic science [1–4]. Research misbehavior includes a broad array of behaviors, some of which may invalidate research results, some that damage trust in science, and others that may deny credit to those to whom credit is due in ways that may hamper their career progression, possibly leading to their exit from the scientific workforce and the loss of highly talented individuals [5]. These behaviors range in “severity” or “seriousness” from research misconduct (fabrication, falsification and plagiarism, henceforth RM) to “lesser” forms of misbehavior usually termed questionable or detrimental research practices (henceforth: QRP) [6]. These behaviors also differ in their level of intentionality and may be just negligent or reckless, or conscious deviations from the standards for good quality research with a purpose other than finding true answers.

Explanations for why researchers misbehave can generally be grouped into three clusters of potentially explanatory factors: those at the level of the individual, factors arising from the organization in which researchers go about their work, and forces that may act upon individual researchers from beyond their immediate workplace - such as the commonly referenced “publish or perish” pressure [7–10].

Examples of individual-related factors are gender or academic rank. Examples of climate factors are perceptions of research-related norms and fairness of supervision, and the quality of resources available to support researchers in their work. Examples of publication system factors are the perceived publication stress among academic researchers and their attitudes towards the current publication system governing academic research.

Previous research has found that male researchers were overrepresented when reviewing RM reports and that junior researchers also seem more likely to report QRPs or RM. In addition, researchers are supposedly more likely to misbehave in a climate where they feel treated unjustly and perceive heavy competition. Lastly, RM and QRPs have been associated with high perceived publication pressure [11–13].

### Objectives

In this paper, we integrate our previously published findings [14–16] that used measurement instruments that are at best proxies for these complex phenomena to see what share of variance in QRPs and RM these three groups of factors account for. We work from the assumption that in a poor-quality research climate with high publication pressure, researchers should be more likely to observe research misbehavior. Our research question is: to what extent can individual, climate and publication factors explain the variance in frequently perceived research misbehaviors?

## Methods

### Study design

We used a cross-sectional survey design.

### Participants

Participants were academic researchers employed at two universities in Amsterdam (Vrije Universiteit Amsterdam and University of Amsterdam) and two academic medical centers (i.e., Amsterdam University Medical Centers, location AMC and VUmc). In order to be eligible for participation, respondents had to be employed in research for at least 1 day per week. We included PhD candidates, as they are formally employed by Dutch institutions. A full description of our recruitment procedure can be found elsewhere [15].

### Variables

The survey questionnaire consisted of three instruments (Survey of Organizational Research Climate, henceforth: SOURCE [17], the revised Publication Pressure Questionnaire, henceforth: PPQr [18], 20 randomly drawn research misbehaviors from a list of 60 QRPs and RM [5]) and three demographic items (gender, academic rank and disciplinary field). For an overview of the different subscales and items that we used as proxies for the individual, climate and publication factors, see Table 1.

**Table 1:** Overview of instruments used in survey questionnaire

Construct of interest	Instrument (reference), ***interpretation***	# items, (# subscales)	Reliability of scores
Individual factors	Gender (male*/female) Academic rank (PhD student*, postdoc or assistant professor, associate or full professor) Disciplinary field (biomedical sciences, natural sciences, social sciences and humanities*)	3	*Not applicable.*
Climate factors	SOURCE [17], *The higher the subscale score, the more positive the perceptions of the research climate.*	28 (7)	Cronbach’s α ranges from .81 to .87
Publication factors	PPQr [18], *The higher the subscale score, the more negative the perceptions of the publication system.*	18 (3)	Cronbach’s α ranges from .75 to .80
Research misbehaviors	List of QRPs and RM [5]	60	Generalizability coefficients for perceived frequency is .80 and .89 for perceived impact, respectively.

* reference category- to ease interpretation, we chose the group with the highest or the lowest score

### Setting

Between May 2017 and July 2017, we conducted a survey study among academic researchers in Amsterdam. We used Qualtrics (Qualtrics, Provo, UT, USA) to design the survey. The survey started after participants indicated informed consent. The survey included three measurement instruments that we previously reported individual results of and here we integrate these findings.

### Study size

We invited the complete population of interest; no specific sample size calculations were made prior to data collection.

### Bias

The greatest source of potential bias in our design is response bias, which is why we sent multiple reminders and advertised our study in university newsletters and on the intranet. Still, the choice to participate in a study related to research integrity and misbehavior is presumably not random.

### Quantitative variables

Explanatory variables are the demographic characteristics of the participant (we refer to these as individual factors, as they regard characteristics of the individual), SOURCE subscales, and PPQr subscales.

Outcome variables are (1) perceived frequency (never observed/observed)^1^ and (2) perceived impact, the product score of perceived frequency and impact on validity that we henceforth denote as perceived impact.^2^ We use perceived impact because focusing on perceived frequency alone may result in a model that explains more trivial trespasses only. We took the square root of this perceived impact score for normalization purposes.

To give the reader an indication of the overall frequency of perceived misbehavior, we calculated percentages of the three possible frequencies. To get a sense of the reliability of our outcome measures, we calculated generalizability coefficients, based on the theory of generalizability developed by Cronbach and colleagues [19]. The generalizability coefficient is a function of variance components and can also be estimated with incomplete data.

### Statistical methods

Each participant responded to 20 items, randomly selected out of a set of 60 items. As a result, participants responded to different sets of items. We applied multilevel logistic regression analysis to the perceived frequency item scores and multilevel linear regression analysis to the perceived impact item scores, with items nested within respondents, and the characteristics of the participants as the higher-level variables. We thus treated the 60 questions about QRPs and RM as “level 1” observations, with those observations nested within respondents, “level 2”. The nesting of observations within level two means that those observations are not independent (in fact, ICCs are 0.17 for Frequency scores and 0.28 for Impact scores) which is why multilevel analyses is appropriate as it is designed to take this non-independence into account, and adjust the standard-errors appropriately to reflect the true “effective” sample size. This application of multilevel models is not yet as common as other applications, such as with student data, where students are the level 1 observations, nested within classrooms (level 2), or such as within-persons repeated measures data, where each time-point provides level 1 measures nested within persons (level 2). But just as multilevel analyses appropriately account for the non-independence of observations in such applications, we used multilevel analyses to account for the non-independence across measures of RM and QRPs^3^ (level 1) within individual respondents (level 2) (for an in-depth explanation, see [20, 21].

Perceived frequency item scores were dichotomized, as the third response option was hardly used (0 = not observed, 1 = observed). The concept of explained variance is not defined in multilevel logistic regression. However, as our application items are first level units and respondents are second level units, the estimated intercept variance represents between-subject variance [20]. We can compare intercept variance in the empty model with intercept variance in models that include explanatory variables, and use unity minus the proportional reduction in intercept variance as an index of explained variance.

Our approach comprised four steps: first, we analyzed the influence of each explanatory variable on the two outcome variables individually. Second, we used a stepwise procedure to assess which cluster of explanatory variables explained most variance (cluster 1, individual factors = gender, academic rank and disciplinary field, cluster 2, climate factors = 7 SOURCE subscales and cluster 3, publication factors = PPQr subscales). Third, we employed a hierarchical model where we consecutively added the explanatory variables in their clusters – starting with cluster 1 – to assess how much cumulative variance was explained. Finally, we inspected the relationships between the different explanatory variables with Pearson’s correlation and regression analyses.

## Results

### Response rate

We obtained 7548 e-mail addresses of active academic researchers in Amsterdam of which 83 were no longer in use. Some researchers explicitly declined participation (*n* = 109) and 1298 researchers completed at least one subscale from the SOURCE, which was sufficient to use their responses in our models, yielding a response rate of 17%.

### Descriptive data

Demographic information can be found in Table 2.

**Table 2:** Participants’ demographic information^a^

**Gender**	
*Male*	441
*Female*	632
**Academic rank**	
*PhD students*	503
*Postdocs and assistant professors*	318
*Associate and full professors*	216
**Disciplinary field**	
*Biomedical sciences*	603
*Natural sciences*	119
*Social Sciences*	242
*Humanities*	109

^a^Two hundred twenty-five participants did not indicate their demographic information or stopped prematurely

### Outcome data

Percentages of each frequency for all 60 QRPs and RM (as well as for the SOURCE and PPQr) can be found in the Additional file 1: appendix.

### Main results

We assessed the association of each explanatory variable with both the perceived frequency measure and the perceived impact measure. An overview of these results can be found in Table 3. Note that these are all separate univariate multilevel regression analyses with a single variable in the model (not corrected for any confounders). Individual factors explain between 0 and 5% of the variance in perceived frequency of research misbehaviors, climate factors explain between 5 and 18% and publication factors explain between 1 and 15% of the variance in frequency of research misbehaviors.

**Table 3:** Explanatory variables for perceived research misbehaviors, univariate analyses

	Perceived frequency of misbehavior				Perceived impact of misbehavior			
Explanatory variable	β	SE^a^	*p-value*^b^	%Index of Expl. Variane^c^	β	SE^a^	*p-value*^b^	%Expl. Variane^c^
***Individual factors***								
**Female** ^vs male^	−.159	.065	.015	.69%	−.009	.021	.651	0%
**Academic rank**				6.06%				1.08%
Postdoc/assistant professor ^vs PhD student^	.469	.072	<.001		.076	.024	.001	
Associate/full professor ^vs PhD student^	.617	.081	<.001		.036	.027	.177	
**Disciplinary field**				.25%				.13%
Biomedical sciences ^vs humanities^	.127	.109	.245		.023	.034	.508	
Natural sciences ^vs humanities^	.110	.139	.430		.034	.044	.442	
Social sciences ^vs humanities^	.035	.121	.771		.048	.038	.209	
***Climate factors***								
**RCR Resources**	−.309	.039	<.001	6.01%	−.079	.012	<.001	3.77%
**Regulatory Quality**	−.463	.050	<.001	5.26%	−.041	.017	.016	.95%
**Departmental Norms**	−.565	.044	<.001	14.67%	−.140	.014	<.001	8.09%
**Integrity Socialization**	−.318	.039	<.001	6.31%	−.072	.012	<.001	3.17%
**Supervisor-supervisee Relations**	−.425	.040	<.001	10.57%	−.098	.013	<.001	5.08%
**Integrity Inhibitors**	−.542	.039	<.001	17.46%	−.156	.012	<.001	12.98%
**Departmental Expectations**	−.319	.037	<.001	7.56%	−.091	.012	<.001	5.77%
***Publication factors***								
**Publication Attitude**	.580	.045	<.001	14.75%	.166	.014	<.001	11.74%
**Publication Resources**	.185	.051	<.001	1.44%	.080	.016	<.001	2.28%
**Publication Stress**	.323	.039	.039	6.39%	.091	.012	<.001	4.92%

^a^standard error of the *β*, ^b^associated *p* value ^c^percentage of explained variance by the independent variable at issue

When using perceived impact as outcome variable, individual factors explain 1% of variance, climate factors between 1 and 13% and finally publication factors explain between 2 and 12% of variance in perceived impact of research misbehaviors.

We added the explanatory variables in their respective clusters and then followed up with a hierarchical model where we consecutively added the clusters, see Table 4. Individual factors as a cluster explain 7% of variance in perceived frequency of research misbehaviors, climate factors as a cluster explain 22% of variance and publication factors as a cluster explain 16% of variance in perceived frequency of research misbehaviors. Individual factors as a cluster explain 1% of variance in perceived impact of research misbehavior, the cluster of climate factors explains 14% and the cluster of publication factors explains 12% in variance of perceived impact of research misbehaviors.

**Table 4:** Explained variance of clusters of factors using hierarchical mixed modelling

	Perceived frequency of misbehavior				Perceived impact of misbehavior			
*Clusters added*^*1*^	*Index of expl. var*^*0*^	Cum*.explained variance*^*1*^	*Diff. model fit*^*2*^ *(df)*	*Signif. model fit*^*3*^	*Expl. var*^*0*^	Cum*. explained variance*^*1*^	*Diff. model fit*^*2*^ *(df)*	*Signif. model fit*^*3*^
Individual factors^a^	6.74%	6.74%	74.1 (*6*)	<.001	1.18%	1.18%	18.1 (*6*)	.001 < *p* < .01
Climate factors^b^	22.22%	31.64%	358.2 (*7*)	<.001	14.10%	15.66%	205.7 (*7*)	<.001
Publication factors^c^	15.85%	34.21%^*^	32.5 (*3*)	<.001	12.28%	18.42%^*^	37.6 (*3*)	<.001

^0^ = this is the explained variance when *only* one group of factors is analyzed, i.e. just the climate factors explain 22.22% of variance perceived frequency of research misbehaviors

^1^ = the explained variance here is the cumulatively explained variance. Since the models are hierarchical, factors are added consecutively, i.e. the explained variance is 31.64% when both individual as well as climate factors are added to the model

^2^ = difference in model fit, model fit here is the difference between the − 2 Log likelihood of the previous model, i.e. 74 is the difference between the intercept-only model and the model with individual factors added, etc.

^3^ = significance of model fit is contrasted with the previous model; the row above or a model with no parameters (vs. individual factors)

^a^ = gender, academic rank and disciplinary field, ^b^ = SOURCE subscales, ^c^ = PPQr subscales

* Note that the explained total is less than the sum of its parts because there is some overlap in the variance that publication and climate factors explain

We followed up with a hierarchical model where we consecutively added the clusters. The clusters of individual factors and climate factors combined explain 32% of the variance in perceived frequency of research misbehaviors. Adding all three clusters to the model, hence including publication factors, explains 34% of the variance in perceived frequency of research misbehaviors. When using perceived impact as the outcome variable, individual and climate clusters combined explain 16% of variance in variance of perceived impact of research misbehaviors. Finally, adding all three clusters to the model explains 18% of variance of perceived impact of research misbehaviors.

### Other analyses

Note that publication factors explain little additional variance when individual and climate factors are already in the model, which prompts questions about the relationship between the different explanatory variables. To assess why adding publication factors last to the model had only a marginal effect on the cumulative increase in variance, we calculated Pearson correlation coefficients between the individual factors and the publication factors and between the climate factors and publication factors (see Additional file 1: appendix). We already looked into the effects of individual factors on publication factors in another paper [1]. To see the additional effects of climate factors on publication factors, we ran further regression analyses (see Additional file 1: appendix). Overall, we found that the more positive a participant’s perception of the research climate, the less negative that participant’s perception of the publication system.

## Discussion

### Key results

We investigated the extent to which variances in research misbehavior can be explained by individual, climate and publication factors. Overall, individual, climate and publication factors combined explain 34% of variance in perceived frequency of research misbehavior and 18% in perceived impact of research misbehavior. The cluster accounting for the greatest percentage of explained variance is the research climate, 22 and 14% in perceived frequency and perceived impact of research misbehavior, respectively. Publication pressure is the second greatest explanatory variable, accounting for 16% of variance in perceived frequency and 12% of variance in perceived impact of research misbehavior. Individual factors are the smallest cluster, explaining 7% of variance in perceived frequency and 1% in perceived impact.

### Interpretation

We found academic rank to play the greatest role within the cluster of individual factors. Previous research coined explanations for the association between academic rank and research misbehavior including the idea that junior researchers are less familiar with responsible research practices [8], or, when under pressure to perform, they would potentially compromise their ethics [16]. However, our results indicate that senior researchers observed significantly more research misbehavior. Hence, perhaps junior researchers are more honest in their self-reporting but when asked about the behavior of others, senior researchers are equally critical of their colleagues.

We found no effect of gender and in fact the influence of individual variables (such as gender) for research misbehavior has received criticism. For example, Kaatz, Vogelman & Carnes [22] pointed out that males being overrepresented among those found guilty of misconduct and evidence from other areas found men more likely to commit fraud, are insufficient to conclude that male researchers would be more likely to engage in research misconduct. Besides, Dalton & Ortegren [23] found that the consistent finding that women respond more ethically than men was greatly reduced when controlling for social desirability. The authors note that this does not indicate males and females to respond equally ethical, but simply that the differences in ethical behavior may be smaller than initially assumed.

We found the cluster of climate factors to have the greatest share in explaining research misbehavior, which is similar to Crain and colleagues [24] who found that especially the subscale Integrity Inhibitors subscale (a scale that measures the degree to which integrity inhibiting factors are present, such as the pressure to obtain funding and whether there is suspicion among researchers) was strongly related to engaging in research misbehavior in their sample of US scientists. A high score on the Departmental Norms (the extent to which researchers value norms regarding scholarly integrity in research, such as honesty) subscale was negatively associated with engaging in research misbehavior. When reviewing the individual subscale effects in our study, these two subscale scores are most strongly associated with perceived frequency as well as with perceived impact. Bearing in mind that we focused on perceptions of engagement in research misbehavior by others in the direct environment and not on research misbehavior by the respondent him- or herself, we still think it is reasonable to believe that we observed a similar pattern. In addition, using a large bibliographic sample based on retracted papers, Fanelli, Costas and Larivière [25] reported that academic culture affects research integrity, again emphasizing the importance of this cluster.

Broadly speaking, the relationship we observed aligns with existing literature that investigates unethical behavior in organizations [26]. A meta-analysis by Martin and Cullen [27] found that unethical behavior (among which they considered lying, cheating and falsifying reports) was associated with what is called an instrumental climate where individual behavior is primarily motivated by self-interest [28]. Related, Gorsira et al. [29] found that when employees perceive their work climates to be more ethical, they were less likely to engage in corrupt behavior and vice versa.

Maggio and colleagues [12] used the previous version of the Publication Pressure Questionnaire and found publication pressure to account for 10% of variance of self-reported research misbehavior among researchers in health professions’ education. This is similar to our findings, although the authors focused on self-reported misbehaviors, whereas we focused on perceptions of engagement in research misbehavior by others in the direct environment. In addition, we used a slightly different set of research misbehaviors and we have investigated researchers from other disciplinary fields as well. Nevertheless, both study results indicate that in an environment where perceived publication pressure is high, the likelihood of researchers reporting research misbehavior will be larger compared to an environment with low publication pressure.

Holtfreter and colleagues [29] used a list of criminological factors that have been associated with research misconduct and asked academic researchers in the US to indicate which factor they thought contributed most to research misconduct. Regardless of their disciplinary field, researchers reported that the stress and strain to perform (among which was the pressure to publish) was the main cause for research misconduct. Holtfreter and colleagues only distinguished two clusters of factors: ‘bad apples’ (similar to our individual factors) and ‘bad barrels’, comprising both climate and publication factors. That said, the stress and strain items are rather similar to our publication pressure items, supporting the idea of publication pressure as a factor contributing to research misconduct.

Note that we do not claim that individual, climate and publication factors are independent. We found, for instance, publication pressure to account for 16% of variance in perceived frequency when added as first variable. However, when climate factors are already in the model, the cumulative increase of explained variance when adding publication pressure is only 2%, which seems intuitive, since it could be that publication factors influence climate factors, such as when increased publication pressure leads to authorship disputes that in turn potentially damage the research climate in particular research groups [13]. A related reasoning could be that publication pressure may arise as a function of how one’s department and departmental expectations for “productivity” are setup, or may arise at a higher organizational level, to the extent that publication expectations are set or influenced by decision makers above the department level.

### Generalizability

Our study’s sample included researchers from different academic disciplines and academic ranks. The findings thus bear relevance to a broad group of academic researchers. Besides, relying on previously validated and repeatedly employed instruments such as the SOURCE [17] and PPQr [18] should substantiate the validity of our findings.

### Limitations

We should acknowledge a number of weaknesses in our study. Firstly, a response rate of 17% is arguably low. That said, it is not lower than other recent surveys that are considered valid [30]. In addition, a low response rate in itself does not indicate a response bias. In another study, we tried to estimate response bias in our sample using a wave analysis and found early responders to be similar to late responders [14]. Also, when looking at demographic characteristics, such as academic rank, our responders seemed similar to the population [15] reducing the concern that our sample is biased, at least with respect to those dimensions. In conclusion, with our response rate, we cannot exclude the possibility of response bias, but we have some reason to believe it should not influence our results substantially.

Secondly, our outcome variables regard perceived misbehavior by others, whereas many studies into misbehavior focus on self-reports of misbehavior by the respondent, including some of the literature we cited. Interestingly, whereas self-reported rates of misbehavior by the respondent have decreased over time, perceptions of the frequency of misbehavior by others have remained more stable [31]. Nevertheless, perceptions of misbehavior measurements may be artificially inflated in situations where various responders have witnessed the same incident. Besides, people are generally more earnest when reporting about others’ misbehavior (and more lenient when it regards their own), also known as the Mohammed Ali effect [31], which could artificially inflate reported perceptions. Hence, our data may overestimate the actual frequency of perceived research misbehavior. Relatedly, as we measured all outcome and explanatory variables through subjective self-report, the correlations between these variables may be inflated by common-method bias [32]. It seems reasonable to say that perceptions carry credible evidence about the ‘true’ prevalence of research misbehavior and its explanatory variables, although surveying perceptions is by no means conclusive.

Thirdly, the assumption that is implicit in our work is that when participants reported on what research misbehaviors they observed in their field of study, they were largely reporting on what they observed in their own research setting. Although we do not think this is an unreasonable assumption, we nevertheless want to acknowledge that we could not test it explicitly in our survey.

Fourthly, it is a characteristic of multiple regression that the more explanatory variables within a cluster, the larger the explained variance. This should be kept in mind, as our clusters have different numbers of explanatory variables within them.

Finally, our results are cross-sectional in nature so we have to refrain from any causal conclusions.

## Conclusions

Our results suggest that researchers’ perceptions of the research climate as well as researchers’ perceptions of publication pressure play a significant role in explaining research misbehavior. Especially the norms that govern research practices in a department and the extent to which integrity inhibiting factors such as suspicion were present, explained a large proportion. Finally, it was not so much a researchers’ publication stress but more their attitudes towards the current publication system that played a substantial role. Note that these proportions of explained variance decreased when using perceived impact as outcome, but the results pattern remained the same. This suggests that efforts to improve departmental norms might have a salutary effect on behavior.

## Supplementary Information

**Additional file 1.** Appendix.

## Data Availability

Data cannot be shared publicly because of participants’ privacy. The pseudonymized personal data is available for research purposes only under a data transfer agreement to ensure that persons authorized to process the personal data have committed themselves to confidentiality and the receiving party agrees that the pseudonymized personal data will only be used for scientific research purposes. To ease the possible exchange of data with fellow researchers, a concept data sharing agreement can be found here: https://osf.io/8jye2. Survey materials can be found here: https://surfdrive.surf.nl/files/index.php/s/rhEAWrUap69jQ6a.

## Footnotes

1 This remains an imperfect measure of how often misbehavior actually occurs, as it relies on whether respondents report observing the behaviour in question.

2 We reasoned that the impact of the misbehavior increases as the behavior is more frequently perceived and is assigned a greater impact on the validity. We used the term impact on the aggregate level another paper, but believe the term perceived impact is more succinct.

3 See Additional file 1: appendix where we juxtapose ordinary regression analysis and multilevel regression analysis.

## References

[1] De Vries R, Anderson MS, Martinson BC (2006). Normal misbehavior: scientists talk about the ethics of research. J Empir Res Hum Res Ethics.

[2] Martinson BC, Anderson MS, de Vries R (2005). Scientists behaving badly. Nature..

[3] Stroebe W, Postmes T, Spears R (2012). Scientific misconduct and the myth of self-correction in science. Perspect Psychol Sci.

[4] Steneck N (2006). Fostering integrity in research: definition, current knowlege, and future directions. Sci Eng Ethics.

[5] Bouter LM, Tijdink J, Axelsen N, Martinson BC, ter Riet G (2016). Ranking major and minor research misbehaviors: results from a survey among participants of four world conferences on research integrity. Res Integr Peer Rev.

[6] Medicine), NASEM (National Academies of Sciences, Engineering A. Fostering Integrity in Research. Washington, D.C.; 2017.

[7] Sovacool BK (2008). Exploring scientific misconduct: isolated individuals, impure institutions, or an inevitable idiom of modern science?. J Bioeth Inq.

[8] Bogner A, Menz W (2006). Science crime. The Korean cloning scandal and the role of ethics. Sci Public Policy.

[9] George SL (2016). Research misconduct and data fraud in clinical trials: prevalence and causal factors. Int J Clin Oncol.

[10] Neill US (2008). Publish or perish, but at what cost?. J Clin Invest.

[11] Tijdink JK, Verbeke R, Smulders YM (2014). Publication pressure and scientific misconduct in medical scientists. J Empir Res Hum Res Ethics..

[12] Maggio L, Dong T, Driessen E, Artino A (2019). Factors associated with scientific misconduct and questionable research practices in health professions education. Perspect Med Educ.

[13] Tijdink JK, Schipper K, Bouter LM, Pont PM, De Jonge J, Smulders YM. How do scientists perceive the current publication culture? A qualitative focus group interview study among Dutch biomedical researchers. BMJ Open. 2016;6(2):e008681. 10.1136/bmjopen-2015-008681 PMC4762115 26888726

[14] Haven TL, Bouter LM, Smulders YM, Tijdink (2019). Perceived publication pressure in Amsterdam –survey of all disciplinary fields and academic ranks. Plos One.

[15] Haven TL, Tijdink JK, Martinson BC, Bouter LM (2019). Perceptions of research integrity climate differ between academic ranks and disciplinary fields: results from a survey among academic researchers in Amsterdam. Plos One.

[16] Haven TL, Tijdink JK, Pasman HR, Widdershoven G, Riet G, Bouter LM (2019). Researchers’ perceptions of research misbehaviours : a mixed methods study among academic researchers in Amsterdam. Res Integr Peer Rev..

[17] Martinson BC, Thrush CR, Crain AL (2013). Development and validation of the survey of organizational research climate (SORC). Sci Eng Ethics.

[18] Haven TL, Tijdink JK, De Goede MEE, Oort F (2019). Personally perceived publication pressure - revising the publication pressure questionnaire (PPQ) by using work stress models. Res Integr Peer Rev..

[19] Cronbach LJ, Nageswari R, Gleser GC (1963). Theory of generalizability: a liberation of reliability theory. Br J Stat Psychol.

[20] Snijders TAB, Bosker RJ (1999). Multilevel analysis: an introduction to basic and advanced multilevel modeling.

[21] Twisk J (2019). Multivariate mixed model analysis. Multivariate mixed model analysis.

[22] Kaatz A, Vogelman PN, Carnes M (2013). Are men more likely than women to commit scientific misconduct? Maybe, maybe not. MBio..

[23] Dalton D, Ortegren M (2011). Gender differences in ethics research: the importance of controlling for the social desirability response Bias. J Bus Ethics.

[24] Crain LA, Martinson BC, Thrush CR, Crain AL, Martinson BC, Thrush CR (2013). Relationships between the survey of organizational research climate (SORC) and self-reported research practices. Sci Eng Ethics.

[25] Fanelli D, Costas R, Larivière V (2015). Misconduct policies, academic culture and career stage, not gender or pressures to publish, affect scientific integrity. PLoS One.

[26] Treviño LK, den Nieuwenboer NA, Kish-Gephart JJ (2014). (un) ethical behavior in organizations. Annu Rev Psychol.

[27] Martin KD, Cullen JB (2006). Continuities and extensions of ethical climate theory: a meta-analytic review. J Bus Ethics.

[28] Simha A, Cullen JB (2012). Ethical climates and their effects on organizational outcomes: implications from the past and prophecies for the future. Acad Manag Perspect.

[29] Gorsira M, Steg L, Denkers A, Huisman W (2018). Corruption in organizations: ethical climate and individual motives. Adm Sci.

[30] Groves R (2006). Nonresponse rates and nonresponse bias in household surveys: what do we know about the linkage between nonresponse rates and nonresponse bias?. Public Opin Q.

[31] Fanelli D (2009). How many scientists fabricate and falsify research? A systematic review and meta-analysis of survey data. Plos One.

[32] Podasakoff PM, MacKenzie SB, Lee J-Y, Podsakoff NP (2003). Common method biases in behavioral research: a critical review of the literature and recommended remedies. J Appl Psychol.

