# Supplementary material for: Explaining variance in perceived research misbehavior: results from a survey among academic researchers in Amsterdam
Source: Res Integr Peer Rev. 2021 May 3;6:7. doi: 10.1186/s41073-021-00110-w (PMC8094603; doi:10.1186/s41073-021-00110-w)
Supplement: Supplementary file 1 — Additional file 1. Appendix. [file 41073_2021_110_MOESM1_ESM.pdf]

Appendix Table of contents

#	Title	p.
1	Percentages of each frequency for the 60 research misconduct and questionable research practice items (as well as the frequencies of each of the answers for the SOURCE and PPQr).	2-5
2	Pearson correlation coefficients between the individual factors and the publication factors.	5
3	Pearson correlation coefficients between the climate factors and publication factors.	6
4	Effects of climate factors on publication factors (regression models).	7-11

1. Percentages of each frequency for the 60 research misconduct and questionable research practice items (as well as the frequencies of each of the answers for the SOURCE and PPQr).

Table 1. Frequencies and percentages of how often each behavior was observed.

	Answer options		
QRPs and RM	**Never,** n (%)	**Once or twice** n (%)	**Three times or more** n (%)
Propose study questions which are clearly irrelevant	196 (55%)	116 (33%)	44 (12%)
Choose a clearly inadequate research design or using evidently unsuitable measurement instruments	204 (59%)	101 (29%)	43 (12%)
Present grossly misleading information in a grant application	230 (86%)	36 (13%)	3 (1%)
Collect more data after noticing that the results are almost statistically significant	228 (65%)	81 (23%)	42 (12%)
Fabricate data	335 (94%)	16 (5%)	4 (1%)
Report on data-driven hypotheses without disclosure	235 (67%)	77 (22%)	37 (11%)
Delete data before performing data analysis without disclosure	302 (85%)	39 (11%)	14 (4%)
Selectively delete data, modify data or add fabricated data after performing initial data-analyses	296 (85%)	44 (13%)	8 (2%)
Perform data-analyses not stated in the study protocol without disclosure	202 (56%)	100 (28%)	56 (16%)
Report an incorrect downwardly rounded p-value	328 (91%)	28 (8%)	5 (1%)
Not report all study protocol-stipulated results	230 (66%)	88 (25%)	29 (9%)
Not publish a valid 'negative' study	192 (54%)	101 (28%)	62 (18%)
Report an unexpected finding as having been hypothesized from the start	176 (49%)	121 (34%)	60 (17%)
Conceal results that contradict earlier findings or convictions	236 (69%)	93 (27%)	15 (4%)
Take no full responsibility for the integrity of the research project and its reports	277 (80%)	54 (16%)	15 (4%)
Refuse to share data with bona fide colleagues	239 (67%)	95 (27%)	20 (6%)
Turn a blind eye to putative breaches of research integrity by others	260 (74%)	78 (22%)	14 (4%)

Refuse to respond to an allegation of a breach of research integrity	335 (94%)	18 (5%)	3 (1%)
Use unpublished ideas or phrases of others without their permission	283 (80%)	57 (16%)	12 (4%)
Use published ideas or phrases of others without referencing	249 (70%)	88 (25%)	19 (5%)
Write no or a clearly inadequate research protocol	213 (60%)	102 (29%)	38 (11%)
Ignore substantial safety risks of the study to participants, workers or environment	323 (90%)	32 (9%)	5 (1%)
Stop data collection earlier than planned because the results are already statistically significant	299 (86%)	40 (11%)	11 (3%)
Not adhere to pertinent laws and regulations	288 (82%)	54 (16%)	8 (2%)
Not report clearly relevant details of study methods	193 (46%)	117 (33%)	46 (13%)
Not report replication problems	266 (76%)	65 (18%)	22 (6%)
Selectively cite to enhance own findings or convictions	124 (35%)	151 (42%)	84 (23%)
Selectively cite to please editors, reviewers or colleagues	110 (32%)	142 (42%)	91 (26%)
Selectively cite or cite own work to improve citation metrics	146 (42%)	123 (36%)	75 (22%)
Let own convictions influence the conclusions substantially	162 (45%)	127 (35%)	73 (20%)
Insufficiently report study flaws and limitations	164 (48%)	126 (37%)	51 (15%)
Spread study results over more papers than needed	147 (42%)	142 (40%)	64 (18%)
Duplicate publication without disclosure	317 (88%)	42 (11%)	3 (1%)
Re-use parts of own publications without referencing	234 (66%)	91 (26%)	29 (8%)
Unfairly review papers, grant applications or colleagues applying for promotion	248 (69%)	78 (22%)	31 (9%)
Review one's own submitted manuscripts	348 (96%)	11 (3%)	5 (1%)
Demand, accept or offer substantial gifts for doing a favour	332 (93%)	15 (4%)	10 (3%)

Insufficiently supervise or mentor junior coworkers	90 (25%)	152 (43%)	113 (32%)
Gross unfairness to collaborators	237 (68%)	85 (24%)	26 (8%)
Submit or resubmit a paper or grant application without consent from all authors	257 (70%)	83 (23%)	24 (7%)
Ignore substantial risks of the expected findings for society or environment	319 (94%)	17 (5%)	4 (1%)
Importantly change the research design during the study without disclosure	247 (70%)	86 (24%)	21 (6%)
Give insufficient attention to the equipment, skills or expertise which are essential to perform the study	166 (48%)	129 (38%)	49 (14%)
Inadequately handle or store data or materials	195 (55%)	115 (32%)	46 (13%)
Keep inadequate notes of the research process	199 (33%)	165 (46%)	73 (21%)
Ignore basic principles of quality assurance	197 (55%)	128 (36%)	31 (9%)
Re-use of previously published data without disclosure	299 (85%)	44 (12%)	9 (3%)
Modify the results or conclusions of a study due to pressure of a sponsor	330 (91%)	30 (9%)	1 (0%)
Failure to disclose a sponsor of the study	316 (90%)	36 (10%)	1 (0%)
Failure to disclose a relevant financial or intellectual conflict of interest	304 (85%)	48 (13%)	6 (2%)
Handle existing conflicts of interest inadequately	247 (71%)	80 (23%)	19 (6%)
Communicate results to the general public before a peer reviewed publication is available	177 (49%)	102 (29%)	79 (22%)
Deliberately communicate findings inaccurately in the media or during presentations	280 (76%)	62 (17%)	26 (7%)
Make no clear distinction between personal views and professional comments	163 (48%)	124 (37%)	51 (15%)
Add an author who doesn't qualify for authorship	110 (31%)	151 (42%)	92 (27%)
Demand or accept an authorship without significant contribution	88 (25%)	144 (42%)	114 (33%)
Omit a contributor who deserves authorship	234 (66%)	105 (30%)	13 (4%)

Not acknowledge contributors who do not qualify for authorship	221 (65%)	96 (28%)	25 (7%)
Not ask permission from contributors for the wording of the acknowledgement	244 (69%)	69 (19%)	44 (12%)
Not share reviewers' comments with all co-authors	273 (77%)	49 (14%)	31 (9%)

Table 2. Frequencies of how respondents scored a particular answer option on the PPQr.

	**Subscales PPQr**		
	**Stress** *n, (%)*	**Attitude** *n, (%)*	**Resources** *n, (%)*
**Answer options[1]**			
1 "*Totally disagree*"	16, (2%)	1, (0%)	0
2	166, (15%)	60, (5%)	28, (3%)
3	427, (40%)	344, (32%)	233, (22%)
4	398, (37%)	555, (52%)	636, (60%)
5 "*Totally agree*"	68, (6%)	116, (11%)	166, (16%)

[1] Rounded off to integer values.

Table 3. Frequencies of how respondents scored a particular answer option on the SOURCE.

	**Answer options[1]**, *n* (%)				
**SOURCE Subscales**	1 "*not at all*"	2	3	4	5 "*completely*"
Departmental norms	6 (.5%)	48 (4%)	257 (22%)	707 (60%)	164 (13.5%)
Regulatory quality	11 (1%)	55 (6%)	272 (32%)	462 (53%)	70 (8%)
RCR resources	49 (3%)	205 (18%)	530 (40%)	460 (35%)	54 (4%)
Integrity inhibitors (*reverse coded*)	196 (16%)	593 (49%)	326 (27%)	92 (7.5%)	6 (.5%)
Socialization activities	28 (2%)	221 (18%)	501 (41%)	418 (35%)	48 (4%)
Supervisor-supervisee relations	7 (.6%)	55 (5%)	235 (20%)	675 (58.4%)	183 (16%)
Departmental expectations	28 (2%)	157 (13%)	414 (34%)	538 (45%)	72 (6%)

[1] *Rounded off to integer values*

2. Pearson correlation coefficients between the individual factors and the publication factors.

Table 1. Correlations between publication factors and individual factors.

	**Gender (female vs male)**	**PhD students (vs other)**	**Postdoc & Asis. Professor (vs other)**	**Associate & Full Professor**	**Biomedical sciences**	**Natural sciences**	**Social sciences**	**Humanities**
**Negative Publication Attitude**	.01	.01	.105	-.13	.02	-.04	.01	.00
**Lack of Publication Resources**	.17	.36	-.09	-.34	.06	-.10	.02	-.03
**Publication Stress**	.12	-.05	.16	-.12	-.09	-.04	.07	.09

3. Pearson correlation coefficients between the climate factors and publication factors.

Table 1. Correlations between publication factors and climate factors[1].

	RCR Resources	Regulatory Quality	Departmental Norms	Integrity Socialisation	Supervisor-supervisee relations	Integrity Inhibitors	Departmental Expectations
**Negative Publication Attitude**	-.38	-.29	-.37	-.41	-.40	-.53	-.45
**Lack of Publication Resources**	-.32	-.24	-.36	-41	-.48	-.44	-.38
**Publication Stress**	-.31	-.27	-.29	-27	-.40	-.40	-.44

[1] Note that higher SOURCE subscale scores (top row) indicate more positive organizational research climate, whereas higher PPQr sxubscale scores (first column) indicate more negative publication attitudes.

4. Effects of climate factors on publication factors (regression models).

When we investigated the additional effects of individual factors, climate factors, and publication factors on perceived misbehaviours, it was apparent that jointly these factors explain less variance than the sum of each separately. This prompted us to investigate the relationship between these explanatory variables in-depth. The implicit assumption in our hierarchical regression analysis was that individual factors functioned as background factors and affect how the research climate is perceived. The climate factors, in turn, might explain the publication factors -- after all, in a research climate where competition and suspicion are high, researchers may experience more publication pressure, which in turn can increase the chance of researchers engaging in (and thereby other researchers observing) research misbehaviour.
We therefore conducted additional regression analyses with individual factors and climate factors as explanatory variables and the publication factors as outcomes. We did this, upon request of this journal's editor, in three different ways. First, we conducted standard linear regression analysis [Table 1]. Second, to show that multilevel regression analyses of the publication factors give the same results as standard regression analyses, we conducted linear multilevel regression analysis with complete data [Table 2]. Finally, to show that multilevel regression analyses give the same results as standard regression analyses, even if two thirds of all data are randomly omitted[1] (similar to our main analyses, where respondents were presented with a random subset of 20 out of 60 Questionable Research Practices and Research Misconduct items, see Tables 3 and 4 in the manuscript), we conducted linear multilevel regression analyses with the randomly remaining one third of our observations [Table 3].

Table 1. Effects of individual and climate factors on publication factors (publication factors were measured using 3 subscales that consisted of 6 items each), standard linear regression analyses.

	**Attitude**				**Resources**				**Stress**			
	**α, GC(rel), GC(abs)**				**α, GC(rel), GC(abs)**				**α, GC(rel), GC(abs)**			
	.776, .776, .735				.754, .757, .743				.801, .801, .765			
**Explanatory variable**	β	SE[a]	*p-value*[b]	Expl. Var (%)	β	SE[a]	*p-value*[b]	Expl. Var (%)	β	SE[a]	*p-value*[b]	Expl. Var (%)
Individual factors												
**Female** vs male	.022	.062	.723	0.0	.344	.062	<.001	2.9	.235	.062	<.001	1.3
**Academic rank**				2.1				16.2				3.1

[1] *We used the random number generator function in SPSS (with 123456 as the random seed number).*

Postdoc/assistant professor vs PhD students	.148	.071	.038		-.514	.066	<.001		.298	.071	<.001	
Associate/full professor vs PhD students	-.266	.081	.001		-1.029	.075	<.001		-.184	.080	.023	
**Disciplinary field**				0.2				1.1				1.5
Biomedical sciences vs humanities	.032	.104	.761		.130	.106	.218		-.332	.103	.001	
Natural sciences vs humanities	-.111	.133	.404		-.195	.134	.147		-.381	.132	.004	
Social sciences vs humanities	.029	.115	.799		.124	.117	.298		-.128	.115	.264	
Climate factors												
**RCR Resources**	-.386	.030	<.001	14.4	-.338	.030	<.001	11.0	-.314	.030	<.001	9.5
**Regulatory Quality**	-.270	.035	<.001	7.7	-.231	.036	<.001	5.8	-.268	.036	<.001	7.4
**Departmental Norms**	-.368	.029	<.001	13.6	-.358	.029	<.001	12.8	-.286	.030	<.001	8.2
**Integrity Socialization**	-.411	.028	<.001	17.2	-.405	.028	<.001	16.5	-.272	.030	<.001	7.5
**Supervisor-supervisee Relations**	-.390	.028	<.001	15.7	-.469	.027	<.001	22.6	-.357	.028	<.001	13.3
**Integrity Inhibitors**	-.521	.026	<.001	27.0	-.442	.028	<.001	19.0	-.400	.028	<.001	15.8
**Departmental Expectations**	-.443	.028	<.001	19.4	-.381	.029	<.001	14.4	-.438	.028	<.001	18.9

Analysis Interpretation: Reviewing the explanatory variables, note that, for example, gender plays a role in perceived publication resources and stress, but not in publication attitude. The β of .022 of publication attitude means that female researchers have a .022 more negative attitude towards the publication climate compared to male researchers (publication factors range from 1-5 and the higher the score, the greater the perceived publication pressure). For RCR resources, a one unit increase in RCR resources (the more positive the perception of RCR resources), results in a .386 decrease in publication attitude. The remaining figures can be interpreted in the same manner.

Table 2. Effects of individual and climate factors on publication factors, multilevel linear regression analyses.

	Attitude				Resources				Stress			
	GC(rel), GC(abs)				GC(rel), GC(abs)				GC(rel), GC(abs)			
	.776, .735				.757, .743				.801, .765			
**Explanatory variable**	β	SE[a]	*p-value*[b]	Expl. Var %	β	SE[a]	*p-value*[b]	Expl. Var %	β	SE[a]	*p-value*[b]	Expl. Var %
Individual factors												
**Female** vs male	.012	.035	.723	0.0	.182	.032	<.001	2.7	.154	.040	<.001	1.0
**Academic rank**				1.3				15.4				2.2
Postdoc/assistant professor vs PhD students	.083	.040	.038		-.270	.034	<.001		.194	.046	<.001	
Associate/full professor vs PhD students	-.149	.045	.001		-.533	.039	<.001		-.120	.053	.023	
**Disciplinary field**				0.0				0.8				1.0
Biomedical sciences vs humanities	.018	.058	.761		.058	. 054	.285		-.217	.068	.001	
Natural sciences vs humanities	-.062	.074	.404		-.105	.069	.127		-.249	.086	.004	
Social sciences vs humanities	.016	.064	.799		.053	.060	.376		-.084	.075	.264	
Climate factors												
**RCR Resources**	-.216	.016	<.001	14.6	-.172	.016	<.001	11.2	-.205	.020	<.001	9.3
**Regulatory Quality**	-.151	.020	<.001	10.8	-.117	.018	<.001	11.8	-.175	.023	<.001	8.2
**Departmental Norms**	-.205	.016	<.001	14.8	-.185	.015	<.001	13.3	-.186	.019	<.001	9.8
**Integrity Socialization**	-.230	.016	<.001	18.4	-.208	.015	<.001	16.5	-.178	.019	<.001	8.0
**Supervisor-supervisee Relations**	-.218	.016	<.001	15.8	-.241	.014	<.001	22.2	-.234	.018	<.001	13.9
**Integrity Inhibitors**	-.291	.015	<.001	27.4	-.226	.014	<.001	18.3	-.262	.019	<.001	15.3
**Departmental Expectations**	-.248	.016	<.001	20.0	-.198	.015	<.001	15.1	-.286	.018	<.001	19.2

Table 3. Effects of individual and climate factors on publication factors, multilevel linear regression analyses with 2/3rd of the outcome variable (publication factors) missing at random.

	**Attitude**				**Resources**				**Stress**			
	**GC(rel), GC(abs)**				**GC(rel), GC(abs)**				**GC(rel), GC(abs)**			
	0.569, 0.518				0.510, 0.486				0.583, 0.542			
**Explanatory variable**	β	SE[a]	*p-value*[b]	Expl. var %	β	SE[a]	*p-value*[b]	Expl. var %	β	SE[a]	*p-value*[b]	Expl. var %
Individual factors												
**Female** vs male	.029	.045	.510	1.1	.216	.040	<.001	6.8	.230	.051	<.001	3.0
**Academic rank**				3.0				12.8				3.6
Postdoc/assistant professor vs PhD students	.055	.051	.282		-.300	.045	<.001		.187	.059	.002	
Associate/full professor vs PhD students	-.208	.058	<.001		-.511	.050	<.001		-.170	.067	.011	
**Disciplinary field**				1.1				4.1				2.0
Biomedical sciences vs humanities	-.038	.074	.605		.098	.068	.149		-.163	.088	.064	
Natural sciences vs humanities	-.084	.095	.374		-.077	.088	.382		-.308	.112	.006	
Social sciences vs humanities	-.002	.082	.985		.071	.076	.349		-.051	.097	.601	
Climate factors												
**RCR Resources**	-.215	.022	<.001	9.8	-.169	.020	<.001	11.2	-.209	.025	<.001	7.8
**Regulatory Quality**	-.138	.027	<.001	6.3	-.091	.024	<.001	11.6	-.163	.031	<.001	5.8
**Departmental Norms**	-.207	.021	<.001	10.7	-.211	.020	<.001	14.1	-.190	.025	<.001	7.4
**Integrity Socialization**	-.234	.021	<.001	12.0	-.227	.019	<.001	16.0	-.175	.025	<.001	6.3
**Supervisor-supervisee Relations**	-.200	.021	<.001	9.7	-.251	.018	<.001	19.7	-.213	.024	<.001	9.1
**Integrity Inhibitors**	-.281	.021	<.001	17.1	-.225	.019	<.001	15.2	-.249	.024	<.001	10.2
**Departmental Expectations**	-.229	.021	<.001	11.7	-.192	.019	<.001	13.1	-.296	024	<.001	15.3

We observed approximately the same effects across all three types of regression analyses [Tables 1-3]. The differences between Tables 1 and 2 can be ascribed to the use of different estimation methods (ordinary least squares for standard regression analysis versus restricted

maximum likelihood for multilevel regression analysis) and different missing value management (in the Resources scale). The differences between Tables 2 and 3 are due to randomly removing two thirds of the outcome variable scores but fall well within sampling variance. Obviously, using only one third of our observations increases the standard errors and diminishes statistical power.

Of course, using only one third of our data also decreases the reliability of our measurements, as illustrated by the Generalisability Coefficients (GC). When there are no missing data, GC for relative judgements and Cronbach's alpha are equivalent, but conveniently the GC can also be calculated for incomplete data. GC can be calculated in two ways, for relative [GC(rel)] and for absolute judgements [GC(abs)]. GC for relative judgements concerns whether two researchers rank items in the same way (e.g., if researcher A scores higher on item X than researcher B, is researcher A then also more likely to score higher on item Y than researcher B), whereas GC for absolute judgements requires two researchers to give the same scores (e.g., whether researchers A and B both score '4' on item X). We provided both GC(rel) and GC(abs) in Tables 2 and 3.
